# Biodegradable microneedle patch for delivery of meloxicam for managing pain in cattle

**DOI:** 10.1371/journal.pone.0272169

**Published:** 2022-08-02

**Authors:** David A. Castilla-Casadiego, Katherine A. Miranda-Muñoz, Jesse L. Roberts, Anne D. Crowell, David Gonzalez-Nino, Dipankar Choudhury, Frank O. Aparicio-Solis, Shannon L. Servoss, Adrianne M. Rosales, Gary Prinz, Min Zou, Yuntao Zhang, Johann F. Coetzee, Lauren F. Greenlee, Jeremy Powell, Jorge Almodovar

**Affiliations:** 1 Ralph E. Martin Department of Chemical Engineering, University of Arkansas, 3202 Bell Engineering Center, Fayetteville, Arkansas, United States of America; 2 Mcketta Department of Chemical Engineering, University of Texas at Austin, Austin, Texas, United States of America; 3 Department of Biomedical Engineering, College of Engineering, University of Arkansas, Fayetteville, Arkansas, United States of America; 4 Department of Civil Engineering, University of Arkansas, 4190 Bell Engineering Center, Fayetteville, Arkansas, United States of America; 5 Department of Mechanical Engineering, University of Arkansas, 204 Mechanical Engineering Building, Fayetteville, Arkansas, United States of America; 6 Department of Anatomy and Physiology, College of Veterinary Medicine, Kansas State University, 228 Coles Hall, Manhattan, Kansas, United States of America; 7 Department of Animal Sciences, University of Arkansas, B110 Agriculture, Food and Life Sciences Building, Fayetteville, Arkansas, United States of America; Helsingin Yliopisto, FINLAND

## Abstract

Microneedle patches are a promising source for transdermal diffusion of macromolecules and are designed to painlessly penetrate the skin. In this study, a biodegradable chitosan microneedle patch to deliver meloxicam for managing pain in cattle was tested. The potential of reuse of the polymeric solution to fabricate the patches, optimization of fabrication, morphological analysis of the microneedle patch and analysis of preservation of the chemical composition after sterilization were evaluated. In-vitro analysis consisted of studying in-vitro penetration mechanical properties, compression testing analysis of microneedle patch, and in-vitro drug release analysis. In-vivo studies were performed to analyze the dissolution capability of the microneedle patch. Results regarding the physical characteristics, chemical composition, and mechanical properties confirmed that rheological properties of the chitosan solution, present significant differences over time, demonstrating that reusing the solution on the fourth day results in failure patches. Morphological characteristics and chemical composition studies revealed that the process of sterilization (ethylene oxide gas) needed for implanting the patches into the skin did not affect the properties of microneedle patches. In-vitro studies showed that approximately 33.02 ± 3.88% of the meloxicam was released over 7 days. A full penetration of the microneedles into the skin can be obtained by applying approximately 3.2 N. In-vivo studies demonstrated that microneedle patches were capable of swelling and dissolving, exhibiting a dissolution percentage of more than 50% of the original height of microneedle after 7 days. No abnormal tissue, swelling, or inflammation was observed in the implanted area. The results of this work show that chitosan biodegradable microneedle patches may be useful to deliver meloxicam to improve pain management of cattle with positive effects for commercial manufacturing.

## Introduction

Pain impacts animal welfare with millions of farm animals experiencing painful management procedures each year [1–3]. Livestock may undergo procedures such as dehorning, castration, and tail docking, which, regardless of the method used, have been shown to be painful [4, 5]. Even though these procedures are necessary to reduce the risk of injury to caregivers and pen mates as well as to eliminate the risk of pregnancy and aggressive behavior, they can cause distress, leading to behavioral and neurological changes [6]. The American Veterinary Medical Association, American Association of Bovine Practitioners, and American Association of Swine Veterinarians have all recommend analgesia for animals undergoing painful procedures [3, 7, 8]. In addition, consumers are becoming more concerned for the well-being of food producing animals, causing increased need for producers to consider pain management options [4, 9, 10].

Although awareness of analgesics for pain management is starting to increase, there are many challenges associated with providing analgesia to food animals [4, 11]. These challenges include the reduced number of medications available, short duration of action of the drugs, repeated dosing of medication, delayed onset, costs, and regulations [4]. Currently, local anesthetics and nonsteroidal anti-inflammatory drugs (NSAIDs) such as flunixin and meloxicam have been shown to be one option for on-farm analgesia. Flunixin meglumine is labeled to reduce fever, inflammation, and pain in cattle [4]; however, it only provides a limited duration of activity [12, 13]. Meloxicam has been shown to reduce inflammation and provide analgesia with a half-life (t½) of approximately 27 hours compared to 6 hours for flunixin meglumine [14, 15]. However, meloxicam is typically administered orally to animals in the United States, which is not amenable for livestock [16].

A biodegradable microneedle patch to deliver meloxicam is a promising transdermal drug delivery method for managing pain in cattle. Using a microneedle system would avoid administration of oral pain medication, providing an extended duration of pain mitigation. A microneedle patch is a surface composed of needles uniformly distributed and short enough to avoid causing pain [17–19]. It is a micron-scale device constructed by micro-molding processes useful to deliver vaccines, drugs, or small particulate formulation to the skin [20]. In our recent work, we designed and modeled a chitosan microneedle patch to deliver meloxicam [21], which resulted in being able to present an organized distribution and homogeneous dimension of microneedles with low drug concentration, penetrate cow’s ear cadaver skin, and preserve the chemical composition of chitosan and meloxicam [21]. The objective of the study presented in this manuscript was to evaluate parameters of the microneedle patch fabrication including, the potential of reuse of the polymeric solution to fabricate the patches, optimized fabrication, morphological analysis of microneedle patch with high concentration drug loading, and analysis of preservation of the chemical composition after the sterilization process. Additionally, in-vitro and in-vivo studies were evaluated including, in-vitro penetration mechanical properties and compression testing analysis of microneedle patch, drug release analysis, and in-vivo dissolution capability analysis of microneedle patch. Our results show that microneedle patches provide an in-vitro sustained drug release with approximately 33.02 ± 3.88% of the meloxicam released over 7 days. In-vivo studies revealed that microneedle patches were capable of dissolving in-vivo, exhibiting a dissolution percentage of more than 50% of the original height of microneedle after 7 days.

## Experimental section

### Materials and chitosan solution preparation

Chitosan (85% deacetylated, molecular weight: 1526.464 g/mol), purchased from Alfa Aesar by Thermo Fisher Scientific (cat. no. J64143), was used to prepare the polymeric solution to maintain the drug encapsulated to be released. The solution was prepared by dissolving chitosan powder in 10% (v/v) of acetic acid (99.8%, cat. no. 109088) from Sigma Aldrich in ultrapure water at 18 MΩ·cm obtained from an ultrapure water system directly from tap producing Milli-Q^®^ water (MilliporeSigma^™^ Direct-Q^™^ 3 Tap) at a final concentration of 10% w/v. Briefly, 1 g of chitosan was added to 10 mL of acetic acid at 10% w/v. To decrease the dissolution time, the solution was placed in a heating plate set at 70°C for 2 h. Pharmaceutical standard, meloxicam was purchased from Sigma-Aldrich (St. Luis, MD, USA). Meloxicam-d3 was purchased from Toronto Research Chemicals (North York, ON, Canada). LC/MS grade acetonitrile and formic acid were obtained from Fisher Scientific (Waltham, MA).

### Chitosan/meloxicam microneedles patch fabrication

**Fig 1:**
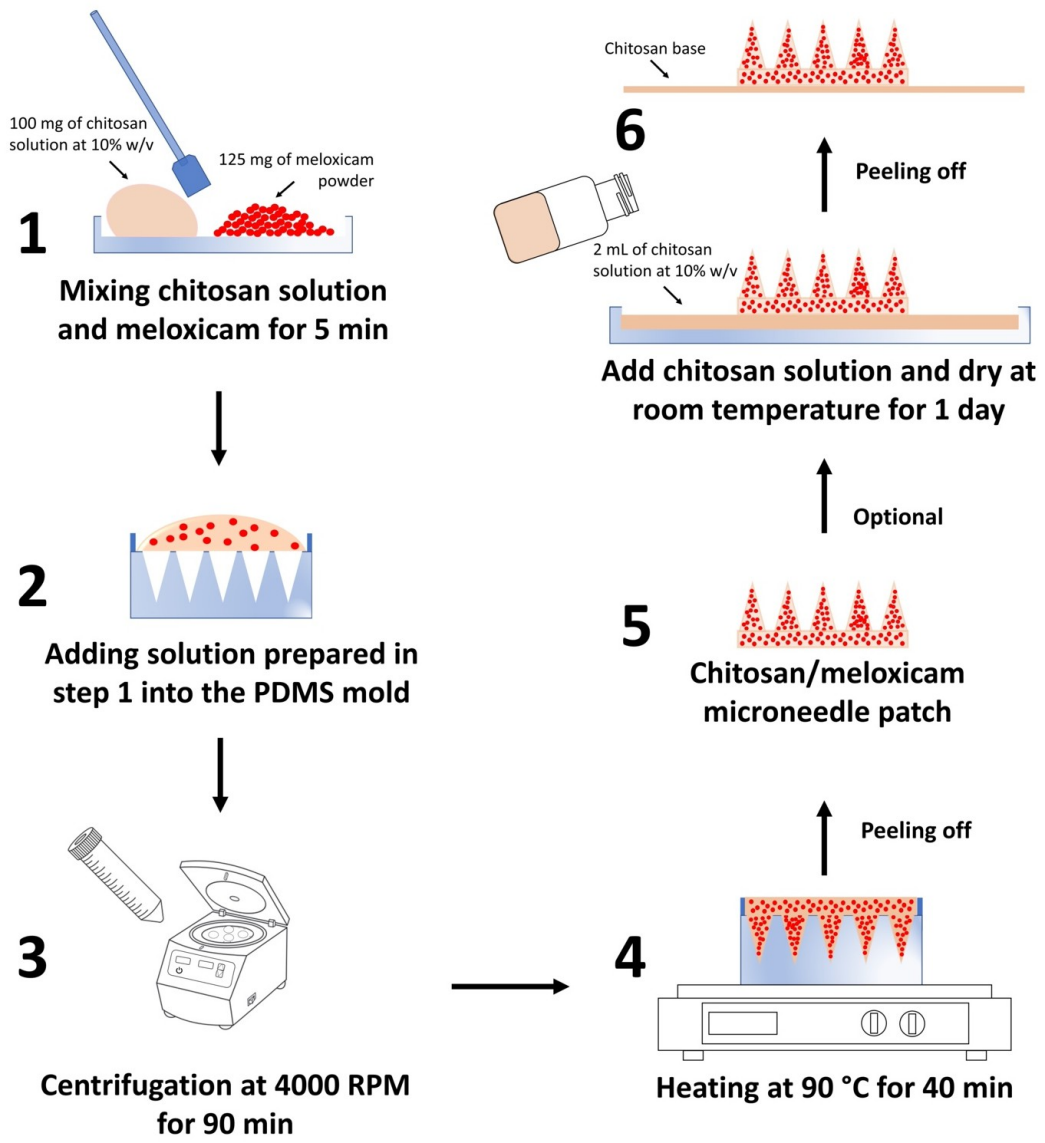
Schematic illustrations of the high concentrated chitosan/meloxicam microneedle patch fabrication process.

The process of fabricating the chitosan/meloxicam microneedle patch, as illustrated in Fig 1, consisted initially of mixing 100 mg or 0.344 mL of chitosan solution with 125 mg of meloxicam (Molecular weight: 351.40 g/mol) purchased from Millipore Sigma (cat. no. PHR1799) for approximately 5 minutes and adding the homogenous mixture onto a (See Fig 1 - step 1 and 2) polydimethylsiloxane (PDMS; Sylgard 184) mold purchased from Micropoint Technologies Pte, Ltd., Singapore (cat. no. ST-05). When the solution was mixed with the drug, the final mixture results in a small, fairly homogeneous, smooth mass with a moldable texture. The dimensions of the mold were as follows: size 8 mm × 8 mm, needle height 600 μm, needle base 300 μm, pitch 250 μm, and array size 15 × 15 (225 microneedles/patch). After that, the mold was put into a 50 mL centrifuge tube with a flat bottom and was centrifuged at 4000 RPM or 2683 g force (RCF) for 90 min using a Hettich ROTOFIX 32 A Cell Culture Centrifuge from VWR (cat. no. 10813–152) (See Fig 1 –step 3) (centrifugation process of the polymer/drug in the mold was monitored every 30 mins). Then, the mold was placed on a hot plate to dry at 50°C for 40 minutes; the mold surface was directly exposed to the hot plate surface, as shown in Fig 1 –step 4. The microneedle patch in the mold was cooled at -20°C for 5 minutes and gently removed from the mold with tweezers (See Fig 1 –step 5). An optional step to generate a chitosan base can be added by spreading 2 mL of chitosan solution onto a flat surface, placing the microneedle patch at the center of the surface, and drying at room temperature for 1 day (See Fig 1 - step 6). Microneedle patches without meloxicam (chitosan microneedle patches) were used as a control for the in-vivo studies and were fabricated as described in our previous publication [21].

### Rheology analysis of chitosan solution

The rheological properties (viscosity, storage modulus, and loss modulus) of the chitosan solution without drug were measured with a TA Instruments Discovery HR-2 rheometer. A cone-and-plate geometry (2° cone angle, 20 mm diameter) was used with a Peltier plate maintained at 25°C. Before beginning measurements, mineral oil was applied to the outer edge of the sample to prevent evaporation. Frequency sweeps were conducted at 1% strain with angular frequencies ranging from 0.01 to 100 rad/s. Shear rate sweeps were performed for shear rates from 0.001 to 100 s^-1^. At each shear rate, the software averaged the data every 10 s. Once three consecutive averages were within 5% of each other, the measurement was recorded. Thus, each data point in the shear rate sweep was collected at a steady state. Measurements of the chitosan solution were made one day after solution preparation and again four days after solution preparation. Before doing measurements, the solution was placed under centrifugation to remove bubbles for 10 mins.

### Morphological analysis of microneedle patch

A TESCAN VEGA3 scanning electron microscope (SEM) operated at 5 keV, and a laser microscope 3D & profile measurement (VHX-7000 Series Digital Microscope) from Keyence were used to analyze the shape, size, consistency, and morphological changes after in-vitro and in-vivo testing of microneedle patches. It was not necessary to coat the microneedle patch with gold to improve conductivity.

### Chemical composition analysis of microneedle patch

A PerkinElmer Frontier FTIR with a Diamond ATR holder within a wavenumber range of 600–1800 cm^−1^ using 4 scans at 4 cm^−1^ resolution was used to collect the infrared spectra of the pure chitosan and chitosan/meloxicam microneedle patches before and after the sterilization process needed for in-vivo studies.

### Mechanical properties analysis of microneedle patch

In-vitro tests were performed to determine the penetration depth of the microneedles into the cow’s ear cadaver skin at different loads. Compression tests were also conducted to determine the load-displacement behavior of the microneedles using the flat end of a stainless-steel pin. Both tests were performed using a tribometer (UMT-2, Bruker, USA) with the testing parameters described in Table 1.

**Table 1:** The in-vitro and compression test parameters.

Experiment type	Name of the step	Time (s)	Loading range (N)
In-vitro penetration test	Constant load for stabilizing	10	0.2
	Linear loading	40	from 0.2 to 3.5
Compression test	Constant load for stabilizing	10	0.2
	Linear loading	40	from 0.2 to 2
	Holding	10	2
	Linear unloading	40	from 2 to 0.2

To assess the skin penetration capability of microneedle patches, ears stored at −80°C (to maintain preservation) were defrosted until reaching room temperature. Before testing, the ear was trimmed and shaved until the hair was removed completely from the skin surface. The first step of the in-vitro test was to stabilize the microneedle on the testing skin at a low load of 0.2 N so that the skin was not penetrated. The stabilization time was 10 s followed by a linear loading step, during which the load was linearly increased from 0.2 to 3.5 N over 40 s, while the penetration depth of the microneedles into the skin was recorded continuously. A similar stabilization step was maintained in the compression test, except the counter-face was the flat end of a 6.35 mm diameter stainless-steel pin. Apart from the stabilization step, the compression test involved a linearly increasing load from 0.2 to 2 N over 40 s, followed by a holding step of 2 N with a duration of 10 s, and finally, an unloading step with linearly decreasing load from 2 to 0.2 N over a duration of 40 s. In both tests, 100 data points were recorded per second.

### In-vitro drug release from the microneedle patch

A chromatography system consisting of a 2695 Separation Module analytical HPLC (Waters, Milford, MA) equipped with a Duragel G C18 150 × 2.1 mm column (Peeke Scientific) and 2489 UV/Vis Detector (Waters, Milford, MA) was used to evaluate the in-vitro drug release from the microneedle patch. A linear gradient of 5 to 95% solvent B (acetonitrile, 0.1% TFA) in A (water, 0.1% TFA) over 30 minutes and a flowrate of 0.2 mL/min was used for the separation of meloxicam. The UV/Vis detector was set to a wavelength of 350 nm, determined using a NanoDrop-2000 UV-Vis Spectrophotometer. To assess the concentration of meloxicam released, microneedle patches were placed in a solution of 20 mL of Dulbecco’s phosphate-buffered saline (DPBS) without Ca^+^ and Mg^+^ (Fisher Scientific, cat. no. 14-190-144) and samples of the drug release were taken at varying time points including, 10 min, 30 min, 1 h, 2 h, 3 h, 6 h, 12 h, 1 day, 2 days, 5 days, 6 days, and 7 days. The amount of meloxicam released in each sample was determined using a calibration curve. Three replicates were analyzed. Results of drug release studies were presented graphically as cumulative percent drug release vs time.

### Animals

Male Holstein steers (n = 6, 100 ± 25 kg body weight) approximately 4 months of age were acquired from a local dairy farm and allowed 14 days to acclimate. Calves were housed in 2m × 2m pens containing 1 calf per pen with concrete flooring and straw bedding. Calves were allowed ad libitum access to water and hay and fed corn gluten pellets twice daily at 1% of body weight.

### In-vivo dissolution mechanism analysis of microneedle patch

To assess in-vivo microneedle dissolution mechanism, patches were manually inserted into cow’s ear by pressing against their backing layer for 3 min to cross the skin’s permeability barrier (the stratum corneum) and promote the absorption of drug under the skin. The ear provides an ideal place for the patch as it allows easy access when cattle are restrained in a squeeze chute. Before the insertion, the ear was trimmed and shaved until the hair was removed completely from the skin surface. Then, to maintain adhesion, patches were covered with three layers of tape consisting of a first layer of an adhesive patch (Estrotect, pbsanimalhealth, cat. no. 17460), a second layer of an elastic tape (ELASTIKON↓, cat. no. 005170), and a final layer of athletic tape (ZONAS↓, cat. no. 005188), as shown in S1 Fig in S1 File. Microneedle patches with and without drug were maintained into the skin for 7 days. Six replicates were analyzed to determine validation of the in-vivo dissolution capability. A TESCAN VEGA3 scanning electron microscope was used to visualize the microneedle patch.

### In-vivo drug diffusion by plasma sample analysis

To assess the transdermal drug diffusion, an in vivo evaluation meloxicam plasma concentration administered from microneedle patches was performed in male dairy calves. The study was carried out at the University of Arkansas Division of Agriculture Research Farm. Experimental treatments included a negative control, a positive control receiving a standard oral dose of meloxicam at 1 mg/kg, as well as two treatments receiving chitosan microneedle patches containing one of two different dosages of meloxicam (2.5 mg/kg and 5.0 mg/kg) on the inside of the calf’s ear. Before placing the microneedle patch on the skin, patches were under a process of sterilization consisting of 24 h under ethylene oxide gas at room temperature. Calves were allocated randomly to treatments to provide 2 calves per each experimental treatment. Blood samples were collected via intravenous jugular catheters (14-gauge x 13cm) at times 0, 20, 40 and 60 minutes; then 2, 4, 7, 10, 24, 30, 48, 72, 96, 144, and 168 hours to evaluate meloxicam levels in blood plasma. The blood plasma samples were evaluated for ultra-performance liquid chromatography-tandem mass spectrometry (UPLC-MS/MS) analysis to evaluate the two dosages of meloxicam levels in the blood plasma over time [22]. Briefly, 100 μL of the bovine plasma sample, 50 μL of the internal standard solution (meloxicam-d3), and 150 μL of 4% phosphoric acid were added to a 48-well nontissue culture-treated plate. Subsequently, the plate was shaken for 20 min and centrifuged at 2,000 × g for 30 min at 20°C. The supernatant was collected onto the Oasis HLB Prime μElution 96-well plate. Following a wash with 300 μL of 5% methanol, the target components were eluted with 50 μL of 90/10 acetonitrile/methanol. 50 μl of 0.1% formic acid was added onto each well of the 96-wells square collection plate before the UPLC-MS/MS analysis. Concentrations of the plasma meloxicam were determined using a Waters Xevo TQ-S triple quadrupole mass spectrometer as described previously with modifications [23]. An acquity UPLC HSS T3 column (1.8 μm, 2.1 × 50 mm) was held at 40°C with eluents composed of mobile phase A (0.1% formic acid) and mobile phase B (0.1% formic acid in acetonitrile). Ions were monitored in the multiple reaction monitoring (MRM) mode with transitions at m/z 352.113 → 115.126 for meloxicam and 355.069 → 155.135 for meloxicam-d3. The calibration curve was linear from 0.05 to 500 ng/mL and accepted when the correlation coefficient exceeded 0.99. The limit of quantification (LOQ) was 0.05 ng/mL. The inter-day accuracy was 107.33%, 110.36%, 99.09%, and 99.88% at concentrations of 5, 10, 50, and 100 ng/mL. The inter-day precision was 2.21%, 1.37%, 0.77%, and 1.36% at concentrations of 5, 10, 50, and 100 ng/mL, respectively.

### Ethics statement

All experiments involving animals were conducted according to the ethical policies and procedures approved by the ethics committee of the Division of Agriculture Institutional Animal Care and Use Committee (Ag-IACUC), University of Arkansas at Fayetteville, United State (Approval no. 21028).

## Results and discussion

### Stability analysis and reuse capability of the chitosan solution

**Fig 2:**
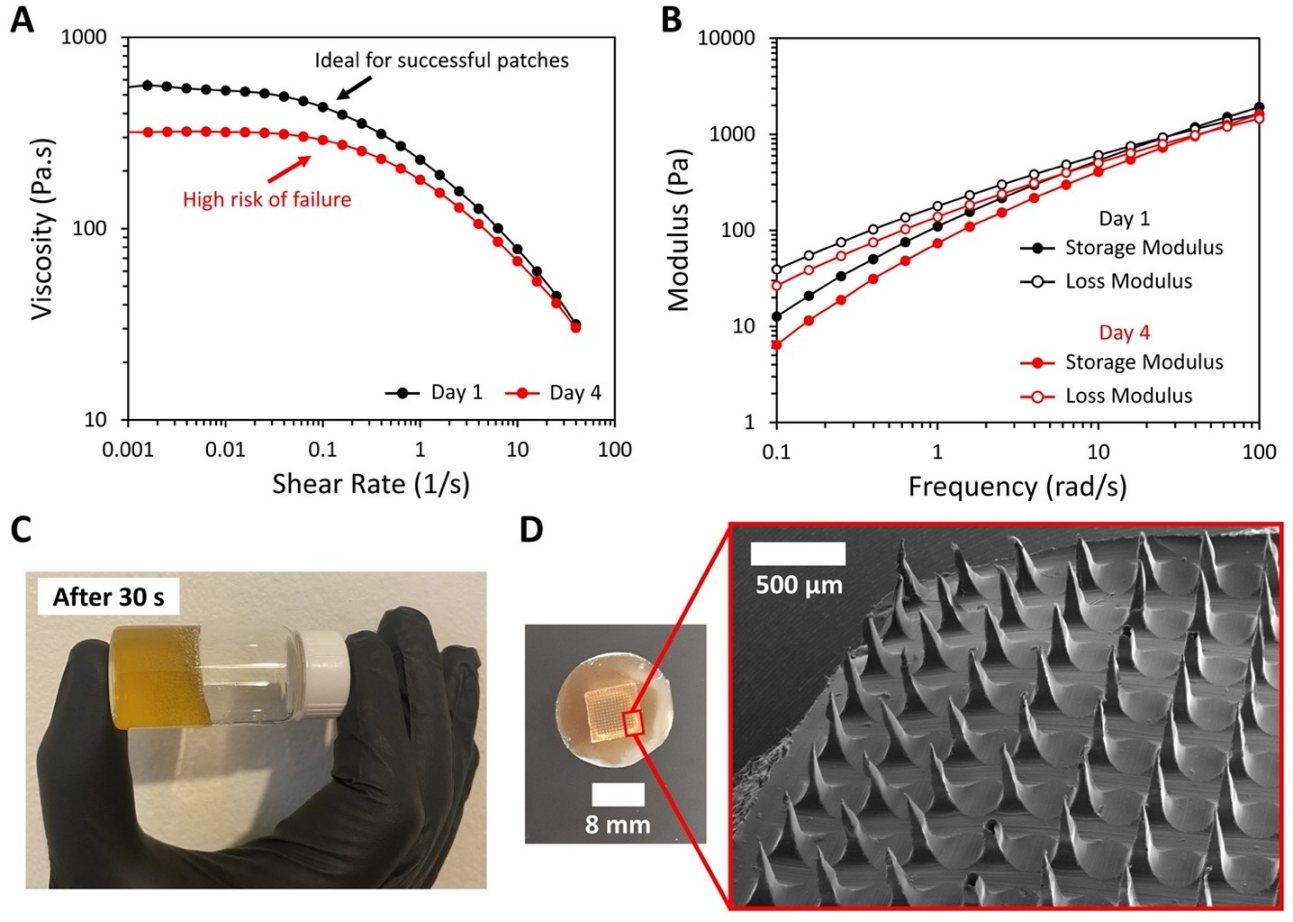
The study of the rheological properties of the chitosan solution confirmed that shear rate sweeps and frequency sweeps both present significant differences over time, demonstrating that reusing the solution on the fourth day results in failure patches. Rheological properties of the chitosan solution at day one and four. A) Viscosity versus shear rate sweeps, B) Storage and loss modulus versus frequency sweeps. C) Visual observation of the viscous chitosan solution on day one. D) Macroscopy picture of a microneedle patch and chitosan microneedle topography by SEM. Data refers to the average of 3 independent measurements.

The stability of the chitosan solution was examined through rheological properties including viscosity, storage modulus, and loss modulus between one and four days after solution preparation. Rheological properties of chitosan solution play an important role in preparing a successful patch to deliver meloxicam. This solution provides support and the capability of incorporating the meloxicam during molding into needle morphology. As a result, it is possible to observe a surface of microneedles loaded with meloxicam when the patch is peeled off to the mold. Therefore, since the successful production of patches is dependent on the rheological properties of chitosan solution, we evaluated the ideal viscosity, storage modulus, and loss modulus of the solution as well as the timescale for solution reuse. The process of fabricating patches demonstrated that successful patches were obtained by reusing the solution for up to three days after preparing the solution. Reusing the solution on the fourth day resulted in patches with deformities including many broken microneedles (See S2A Fig in S1 File). The study of the rheological properties of the chitosan solution confirmed that shear rate sweeps and frequency sweeps both present significant differences in viscosity and storage/loss moduli over time, as shown in Fig 2A and 2B, respectively. Fig 2A shows that for both time points (one and four days, the solution was stored at room temperature), the shear rate sweeps showed a plateau between 0.001 and 0.01 s^-1^. The average viscosity in this region was taken as the zero-shear viscosity (η_0_). Between one and four days after solution preparation, η_0_ decreased. At one day after solution preparation, η_0_ = 540 ± 30 Pa.s, and four days after solution preparation, η_0_ = 320 ± 10 Pa.s, representing a reduction of approximately 40.7%. The drop in viscosity can be attributed to the degradation of the chitosan by hydrolysis, which occurs mainly under the influence of acids [24, 25], such as acetic acid, affecting the degree of degradation and the molecular mass of oligochitosan [26]. Similarly, in Fig 2B the frequency sweep showed that the storage and loss moduli of the solution, which represent a measure of elastic and viscous response of a material, respectively, were lower when comparing both time points, one versus four days. Therefore, results demonstrate that successful patches can be fabricated by reusing the solution for a maximum of 3 days; deformed patches are obtained on the fourth day after solution preparation (See S2A Fig in S1 File). Fig 2C shows a visual observation of the viscous chitosan solution on day one when the vial was held horizontally for approximately 30 s, demonstrating that the solution flows slowly due to its high viscosity. A digital picture of a microneedle patch and SEM image of chitosan microneedles without drug are presented in Fig 2D, revealing that chitosan microneedles without drug have a smooth surface due to the complete dissolution of chitosan in 10% v/v of acetic acid.

### Optimization fabrication and morphological analysis of microneedle patch

**Fig 3:**
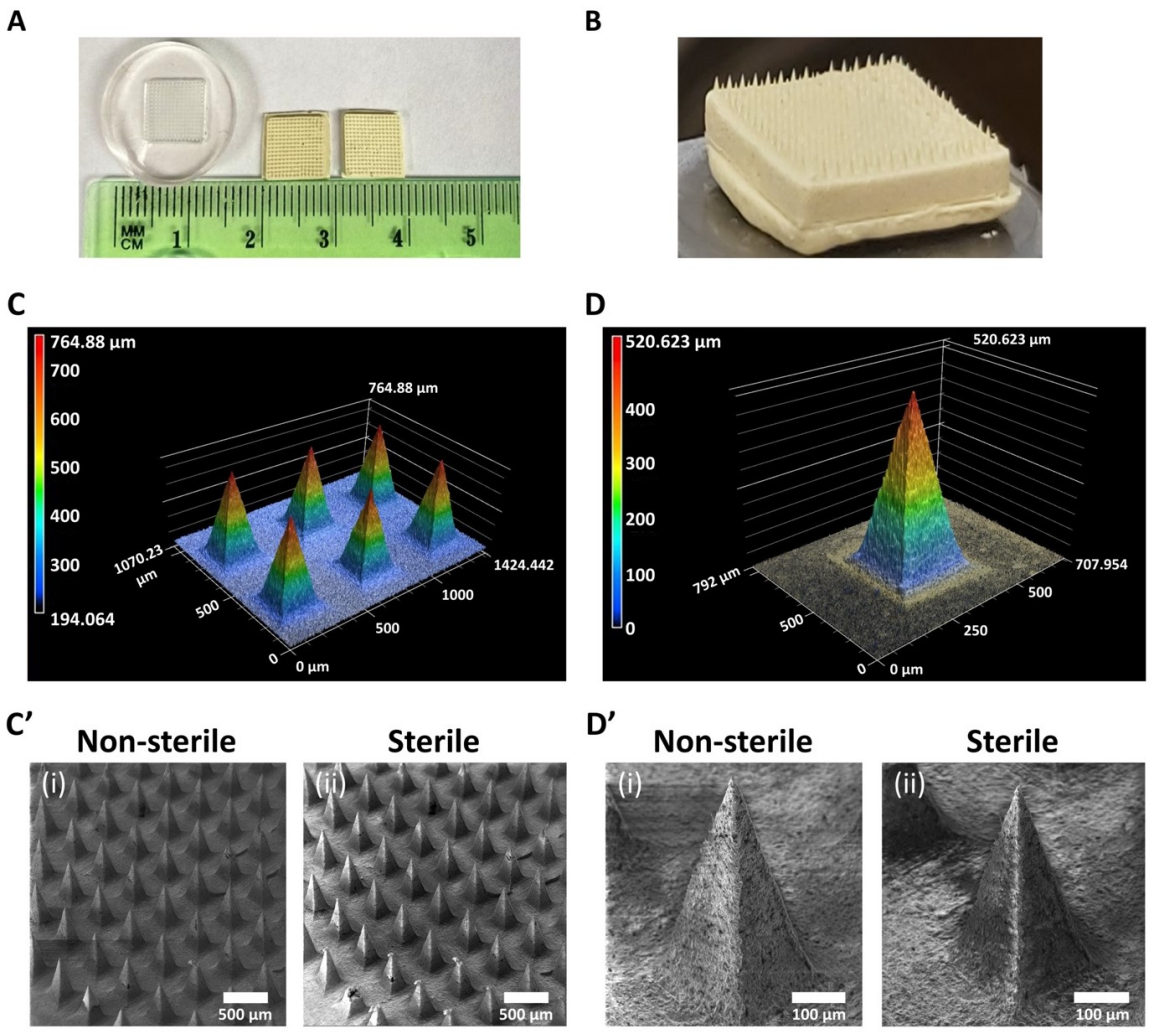
Microscopy characterizations demonstrated that microneedles with high concentration of drug are uniformly organized on the patch surface and preserve their morphological properties after the sterilization process using ethylene oxide gas. Macroscopy view. A) PDMS mold vs. microneedle patches. B) Zoom of microneedle patch surface. Microscopy view. C) 3D laser image of microneedle patch. C’) SEM images of non-sterile (i) and sterile (ii) multiple microneedles patch 3D laser image of microneedle patch. The topography of one microneedle. D) 3D laser image of microneedle patch. D’) SEM images of non-sterile (i) and sterile (ii) microneedle patch.

Physical properties of the microneedle patches with high concentration of meloxicam, including morphology, topography, and distribution were examined before and after ethylene oxide sterilization using SEM and laser microscope 3D & profile measurement. Fig 3 reveals that high concentrated microneedles are uniformly organized on the patch surface and preserve their morphological properties after the sterilization process. Our previous formulation [21] was limited to produce patches with a low concentration of drug of 50 mg/patch, a production time of 3 days per patch, and excessive amount of chitosan solution per patch [21]. Our new formulation reduces the use of multiple patches per cattle and extra stress to the animal because this formulation provides a greater drug concentration of 2.5 times. Results on the optimization of increasing the concertation drug in the patch revealed that by using a mold with the dimensions as follows: size 8 mm × 8 mm, needle height 600 μm, needle base 300 μm, pitch 250 μm, and array size 15 × 15 (225 microneedles/patch), it is possible to fabricate patches with the maximum capacity of 125 mg of meloxicam. Patches loaded with more than 125 mg resulted in defective patches, including broken microneedles and a breakable surface with insufficient capacity to penetrate the skin (See S2B Fig in S1 File). Additionally, the new preparation allowed the reduction of using 10 mL of chitosan solution at 10% (v/v) of acetic acid to 0.344 mL to prepare a single patch. This improvement drastically reduces the exposure of the animal to acetic acid during the drug release treatment. This finding was confirmed through FTIR characterization, which demonstrated that the chemical composition of the concentrated microneedle patch poses insignificant traces of acetic acid, as shown in Preservation of chemical composition of microneedle patch section. In addition, the new formulation showed a favorable time reduction in production of only 3 hour per patch versus 3 days using our initial patch formulation, as previously reported [21].

The new high concentration microneedle patch conserved the dimensions of the PDMS mold, as shown in Fig 3A. Fig 3C and 3D confirm that the height and base length of the microneedles are approximately 600 μm and 300 μm, respectively, with a pitch of 250 μm, matching the dimensions of the mold used. Fig 3B shows the final aspect of the microneedle patch, which shows a patch surface roughness that is due to the presence of meloxicam, which is not soluble in acetic acid. In addition, the patch was similar in color to that of meloxicam due to the high concentration used. Our previous design with low concentration of meloxicam showed the brown characteristic color of chitosan dissolved in acetic acid [21]. This new formulation confirms the production of microneedle patches with low amount of the chitosan solution and insignificant traces of acetic acid. However, the amount of chitosan found in the patch was sufficient to provide support and mold the drug as a uniformly organized surface of microneedles. Fig 3C’ and 3D’ show the topography of the non-sterile and sterile chitosan/meloxicam microneedle patch. This result shows that the topographic characteristics of sterile patches are similar to non-sterile patches, which indicates that the sterilization process does not affect the integrity of the patch.

### Preservation of chemical composition of microneedle patch

**Fig 4:**
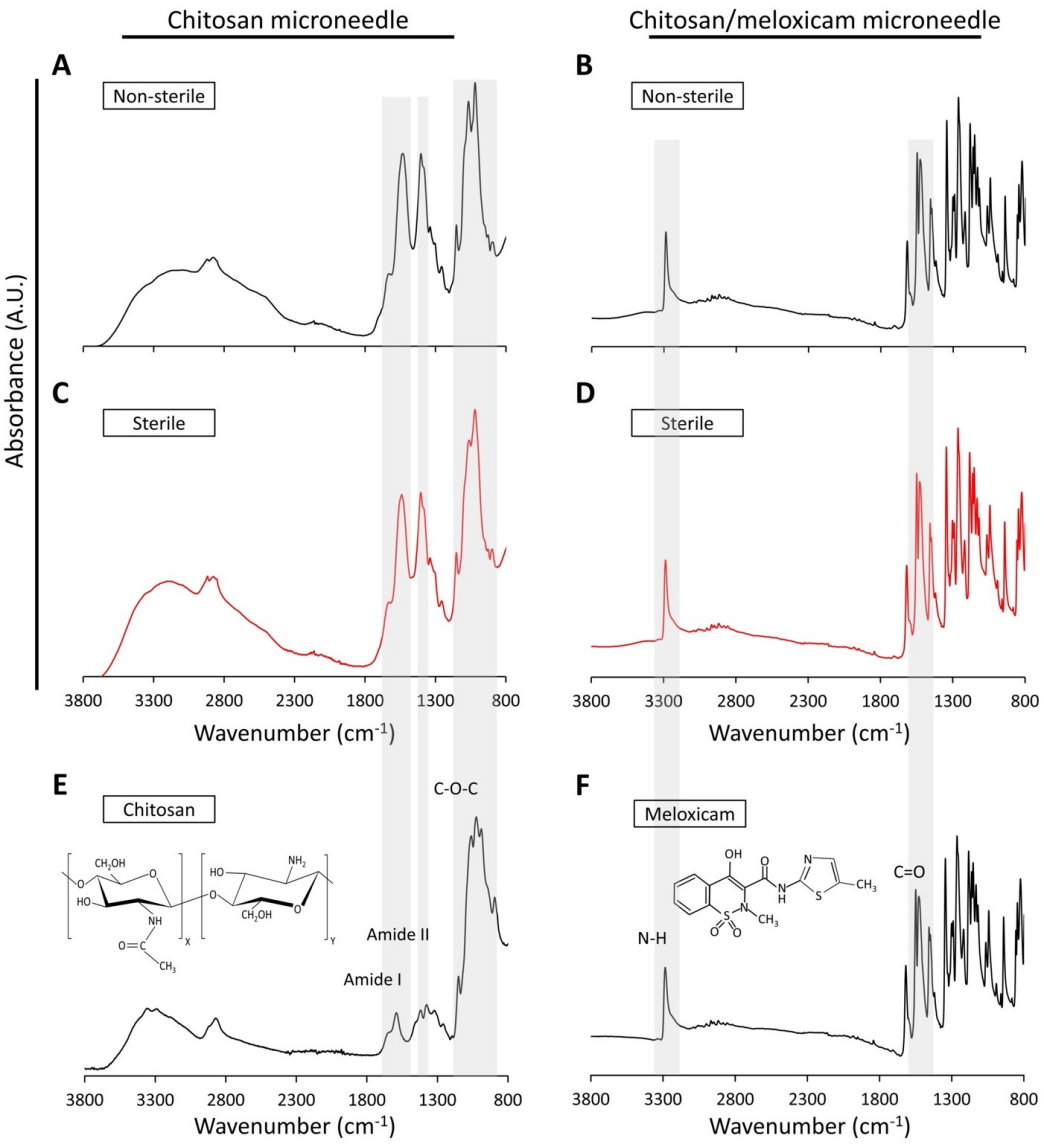
FTIR spectra confirmed that microneedle patches with and without drug preserve the chemical composition of chitosan polymer and meloxicam drug after the sterilization process using ethylene oxide gas. FTIR spectrums of non-sterile and sterile patches. Chitosan microneedle patch. A) Non-sterile, B) Sterile. Chitosan microneedle patch with high concentration of meloxicam. C) Non-sterile, D) Sterile. Spectrums of pure polymer and drug. E) Chitosan, F) Meloxicam.

The chemical composition of microneedle patches with and without drug before and after the sterilization process were examined by FTIR characterization. In addition, spectrums of pure chitosan and meloxicam were obtained. Results confirmed that the chemical composition of the microneedle patches was preserved after treatment with ethylene oxide gas for 24 h (Fig 4). Fig 4E and 4F present the FTIR spectrum and molecular structure of pure chitosan and meloxicam, respectively. Chitosan (Fig 4E) presents characteristic absorption peaks including -OH bond (3435 cm^−1^), C-H stretch (2922 cm^−1^), NH_2_ deformation, amide I (1656 cm^−1^), N-H, N-acetylated residues, amide II (1603 cm^−1^), bridge -O- stretch (1160 cm^−1^), C-O stretch, secondary hydroxyl group (1085 cm^−1^), and C-O stretch, primary hydroxyl group (1030 cm^−1^), as reported in the literature [21, 27–29]. On the other hand, meloxicam shows characteristic absorption peaks located at 3310 cm^−1^ (amine N-H stretch), 3201 cm^−1^ (O-H stretch), 2850 cm^−1^ (aliphatic C-H stretch), 2905 cm^−1^ (aromatic C-H stretch), 1560 cm^−1^ (C = O stretch), 1340 cm^−1^ (aromatic C = C stretch), and 1190 cm^−1^ (aromatic C = C stretch) (Fig 4F) [21, 30]. Comparing FTIR spectrums for chitosan microneedle patches before and after the sterilization process (Fig 4A and 4C) with the pure chitosan (Fig 4E), it is possible to observe that chitosan microneedle patch spectrums exhibit the same characteristic bands of chitosan. The presence of amide I, amide II, and C-O-C stretch indicate that the sterilization process does not generate a significant impact on the chemical composition of the chitosan. Modifications in the spectral data of the chitosan microneedle patches are notable due to traces of acetic acid, indicating that the acetic acid is not completely removed during the drying process.

FTIR spectrums of microneedle patches with meloxicam drug are showed in Fig 4B and 4D. Likewise, patches with meloxicam exhibit the same characteristic bands of meloxicam, as described above. Although patches with drug contain chitosan, FTIR spectrums of patches fabricated with the new formulation (Fig 4B and 4D) containing stronger peaks associated to meloxicam. Those spectrums reveal an extreme similarity to the meloxicam spectrum (Fig 4B, 4D and 4F) because of the lower amount of chitosan solution used to prepare the chitosan/meloxicam patch. In addition, these results confirm that the sterilization process does not alter the chemical composition of the patch, Fig 4B and 4D. Therefore, our formulation allows the fabrication of high concentrated drug into the patches preserving the chemical structure of the meloxicam and using minimum amount of polymeric solution.

### In-vitro mechanical properties: Penetration and compression analysis of microneedle patch

**Fig 5:**
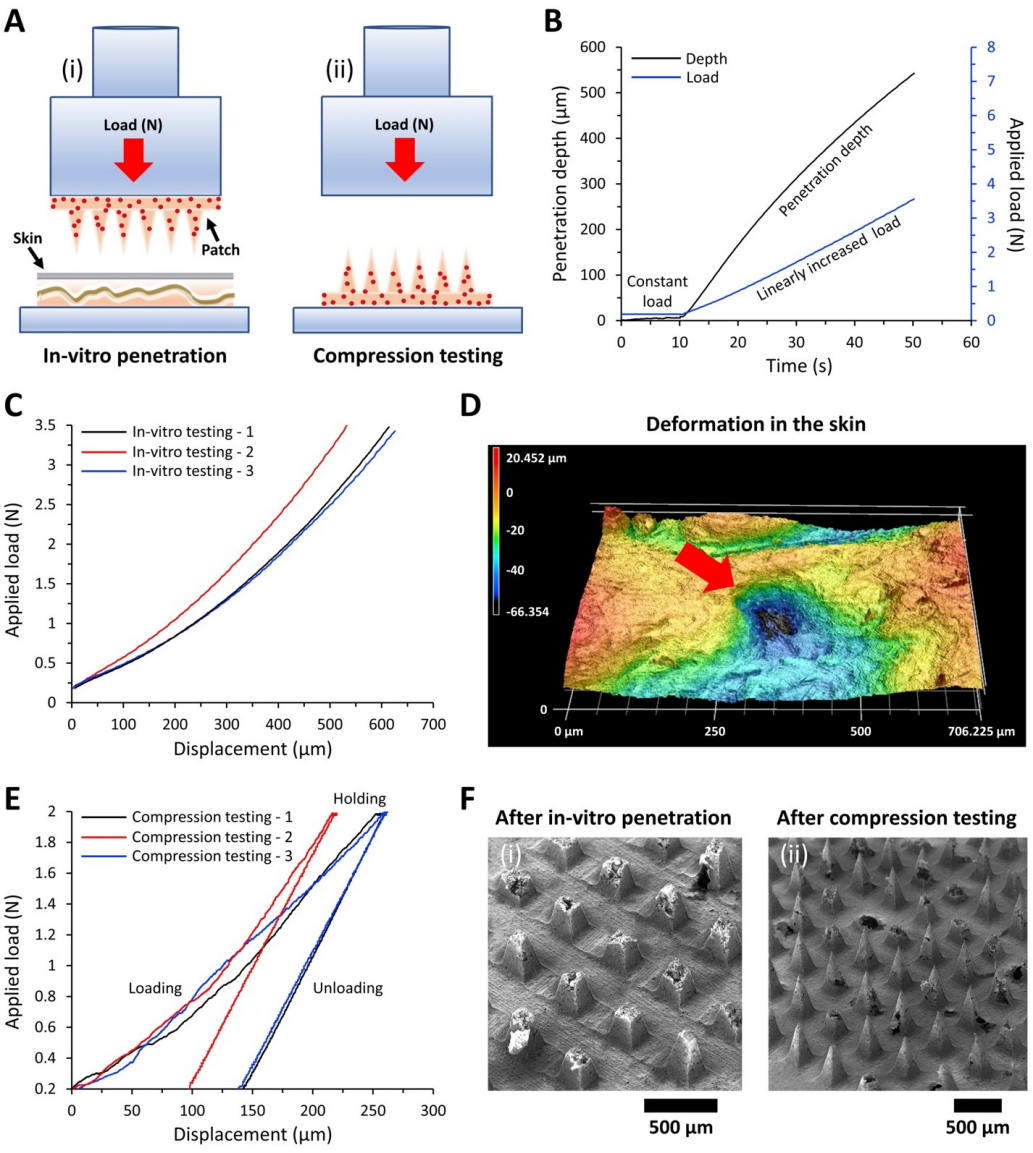
Mechanical property of microneedle patch. A) Schematics of i) in-vitro penetration testing and ii) compression testing set-up, B) applied normal load and measured penetration depth during the in-vitro testing, C) load-displacement relationship obtained from the in-vitro testing (n = three different samples, individually represented in the figure), D) 3D optical topography image of an indent made by one of the microneedles after the microneedle withdrawal, E) the load-displacement relationship obtained from the compression testing (n = three different samples, individually represented in the figure), F) SEM images of the microneedle patch surface after (i) in-vitro penetration and (ii) compression testing.

Mechanical tests were conducted using a tribometer (UMT-2, Bruker, USA) to assess the penetration depth of drug-loaded microneedles into the cadaver skin of cow’s ear at different loads (Fig 5A (i)). These tests were also used to determine the deformation behavior of the microneedles compressed against the flat end of a stainless-steel pin (compression tests) (Fig 5A (ii)). Fig 5B shows an example of the applied normal load and the measured penetration depth during the in-vitro penetration test. Fig 5C shows the load-displacement relationship for three in-vitro penetration tests. At 3.5 N applied load, the microneedles penetrated into the skin almost completely because the indentation depths are similar to the microneedle height. A 3D optical topography image of an indent made by one of the microneedles demonstrated a deformation depth of approximately 66 μm after the microneedle patch was withdrawn from the skin (Fig 5D). Fig 5F (i) shows that the microneedles were broken with the base of the microneedles left on the patch and the tips of the microneedles left inside the skin. This indicates a strong attachment between the microneedle surface and the skin layers, which provides protection against potential peel off from the skin.

The compression test revealed that 2 N applied normal load could compress the microneedles 220–260 μm and cause 100–135 μm permanent deformation (with more than 50% of deformation recovery) after unloading, as shown in Fig 5E. A model of using chitosan microneedles to deliver bovine serum albumin to rat skins was reported by Chen and colleagues [31] using identical dimensions to ours demonstrated that only 0.4 N was required to compress approximately 200 μm of the height of the microneedles. Despite their microneedles exhibited lower mechanical strength than ours, their microneedles were able to penetrate the rat skin and enable in-vivo transdermal delivery of a model protein to the rat skin [31]. Fig 5F (ii) revealed that multiple microneedles were broken, and others presented just slight blunting after the compression testing.

### In-vitro drug release analysis

**Fig 6:**
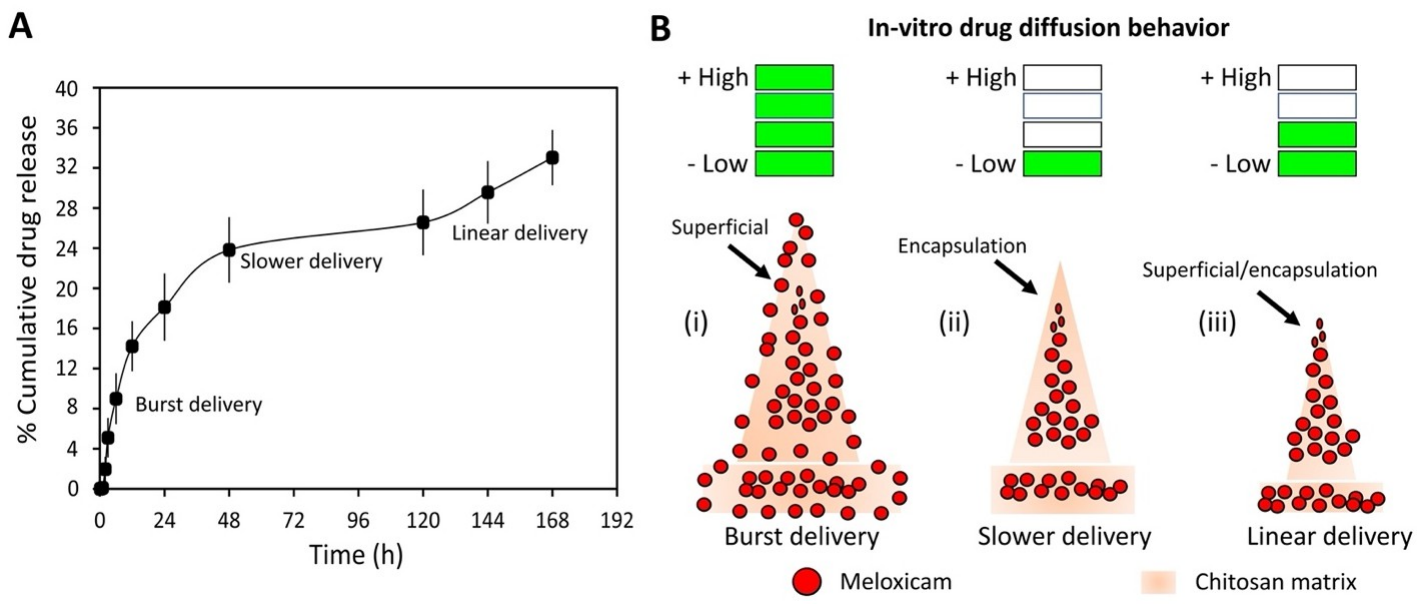
In-vitro drug release analysis reports that microneedles provided a sustained release with approximately 33.02 ± 3.88% of the meloxicam released for 7 days. A) In-vitro % cumulative drug release profile of meloxicam from microneedle patches, B) Schematic representation of drug release (i) Brust delivery, drug on microneedle patch surface, (ii) Slower delivery, drug encapsulated, (iii), linear delivery, drug on microneedle patch surface and encapsulated. Data are presented as the mean ± standard deviation of n = 3 samples.

To evaluate the release of meloxicam from the chitosan microneedle patch, the patch was placed in DPBS solution and subsequently monitored for drug release at room temperature for 7 days. The release of meloxicam from microneedles is described by a triphasic pattern, which consisted of an initial rapid release during the first 2 days followed by a slower release within 2 to 5 days, and finally a linear release behavior (Fig 6A). The initial burst delivery could be associated with the immediate release of meloxicam from the microneedle surfaces (See Fig 6B (i)) [32], which are initially and directly exposed to the DPBS dissolvent used for the in-vitro drug release analysis. The rough surface of the microneedle patch is due to the presence of meloxicam on the surface of the patch, which is not soluble in acetic acid. After 2 days, the patch showed a slower and constant release for three days, which could be attributed to meloxicam that is completely encapsulated in the chitosan matrix, creating a barrier that prevents the rapid release of the drug (See Fig 6B (ii)). Subsequently, after 5 days a linear trend was observed, which could be related to the proportional ratio of chitosan solution and meloxicam in the microneedle patch (See Fig 6B (iii)). As shown in Fig 6A (See S1A-S1B Table in S1 File, in-vitro drug release raw data), our microneedles provided sustained release with approximately 33.02 ± 3.88% of the meloxicam present in one patch, which represents 40.02 ± 4.85 mg of meloxicam released per patch in 7 days. These results demonstrated that our microneedle patch by itself without no modification to control swelling and dissolution has the capability of providing an in-vitro drug release for more than 7 days.

### In-vivo dissolution capability of the microneedle patch

**Fig 7:**
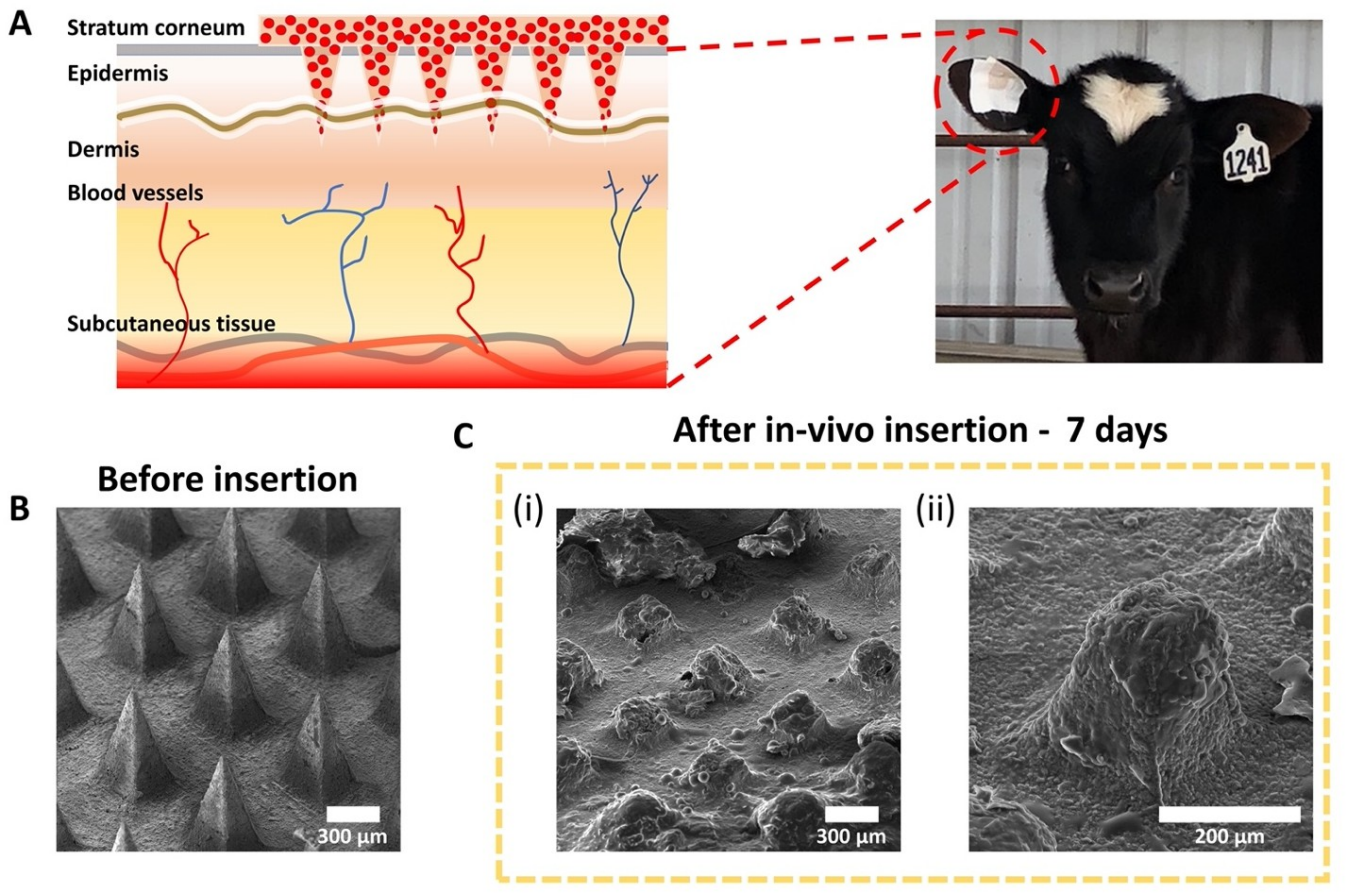
Insertion study demonstrated that microneedle patches were capable of degrading in-vivo. A) Mechanism of transdermal drug delivery of a microneedle patch when penetrating skin layers of cow’s ear. Microneedle patch topography. B) Before in-vivo insertion. C) After 7 days of in-vivo insertion.

To evaluate dissolution capability of the microneedle, the patch was placed in the ear of a healthy calf. Being placed in the ear, there was very little likelihood of the calf being able to lick the patch and possibly remove it. The ear has less hair than other places on the body allowing better adhesion of the patch and absorption of the meloxicam. Fig 7A shows a schematic mechanism of transdermal drug delivery of a microneedle patch when penetrating the skin layers of calf ear. Our patch with microneedles less than 1 mm in height, which are short enough to avoid causing pain [18, 19], were able to physically cross the skin’s permeability barrier (the stratum corneum barrier) promoting the absorption of chitosan and meloxicam under the skin. Briefly, after passing the barrier layer, microneedles are directly placed in the epidermis or upper dermis layer [33]. Chitosan (natural biodegradable biopolymer) [34], which has a degradation dependent on molecular weight, deacetylation degree, polydispersity, purity level, and moisture content [35], is degraded in-vivo by lysozymes or through the process of enzymatic transformation to basic, non-toxic components, such as oligosaccharides that are then excreted or incorporated to glycosaminoglycans and glycoproteins [34]. Other research groups have reported that this biopolymer can be cleared by the kidney depending on its molecular weight (3–50 kDa) or by proteases into fragments for renal clearance if chitosan used has excessive molecular weight (310–600 kDa) [31, 36]. Once the meloxicam begins releasing into the skin layer, the drug goes into systemic circulation through the blood vessels until it reaches the targeted site of action to provide a therapeutic response (Fig 7A).

Fig 7C demonstrates that the in-vivo dissolution of the microneedles was effective. Using SEM, morphological changes of the microneedles before and after in-vivo insertion were assessed, as shown in Fig 7B and 7C, respectively. After 7 days of insertion, results revealed that microneedles experienced more than 50% of dissolution. Fig 7C (i) shows that microneedles present an apparent homogeneous or uniform dissolution, showing a melted surface of less than 300 μm of height due to direct contact with the epidermis and dermis layers of the calf’s ear skin. S3 Fig in S1 File shows different sections of the microneedle patch surface after in-vivo insertion for 7 days. Fig 7C (ii) shows one single microneedle in process to dissolve, demonstrating that the height of the microneedle was less than 300 μm (original height of 600 μm). An important observation is that the base of the patch presents a melted surface, which indicates that the drug is being not only absorbed from the microneedle but also from the base of patch (Fig 7B and 7C). Thus, results indicate that our microneedle patch has the capability to degrade in-vivo when penetrating the epidermis and dermis layer of the skin, which is essential for transdermal drug delivery [33]. Additionally, there was no abnormal tissue, swelling or inflammation noted in the area where the patch was inserted.

### In-vivo transdermal drug diffusion of meloxicam

**Fig 8:**
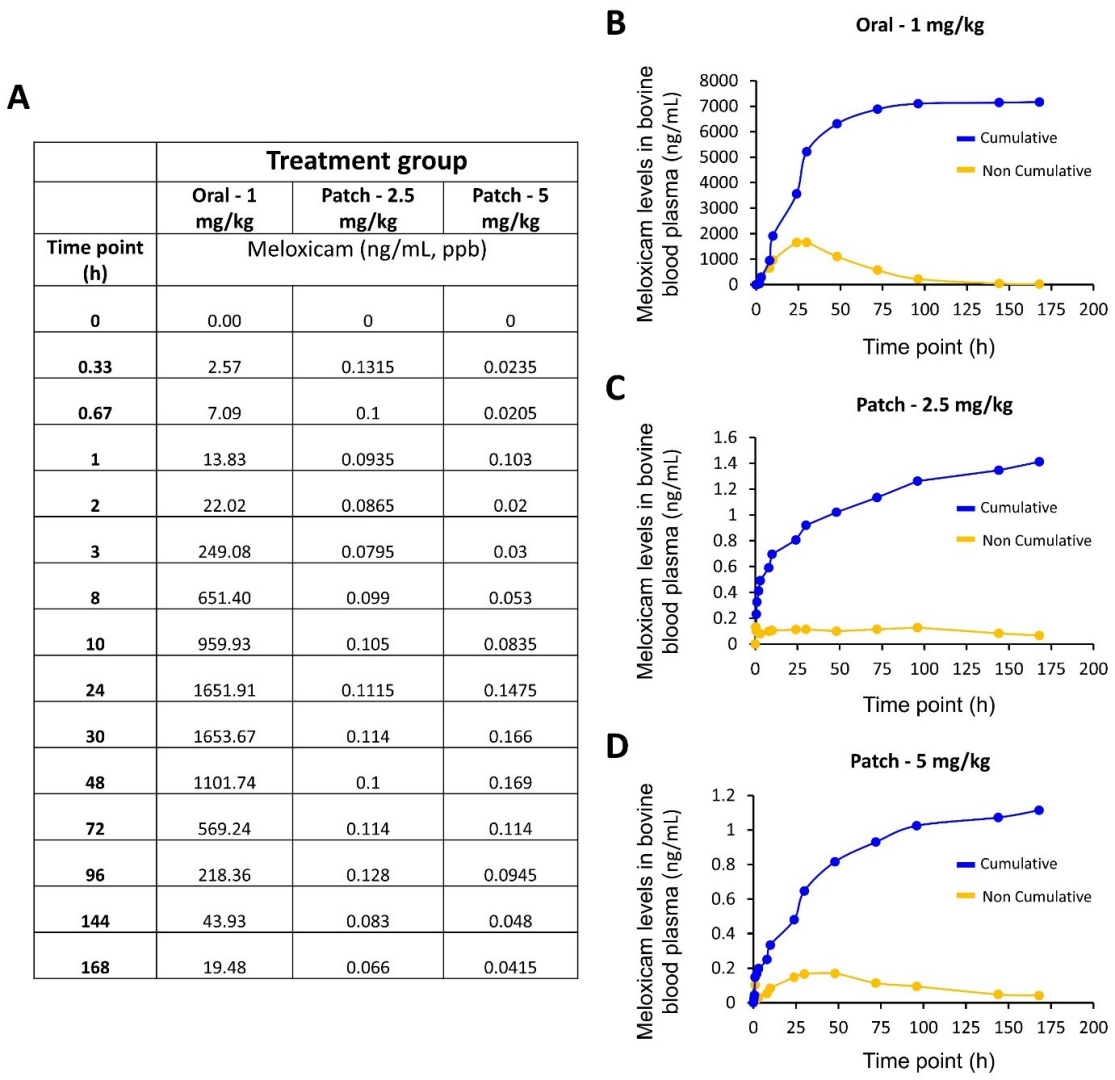
Meloxicam levels in bovine blood plasma. A) Raw data (average), B) Oral -1 mg/kg, C) Patch -2.5 mg/kg, D) patch—5 mg/kg. Data are presented as the mean ± standard deviation of n = 2 samples per condition.

For the In-vivo transdermal drug diffusion study, chitosan microneedle patches containing one of two different dosages of meloxicam (2.5 mg/kg and 5.0 mg/kg) were placed on the inside of the cow’s left ear. To evaluate meloxicam levels in blood plasma samples at times 0, 20, 40 and 60 minutes; then 2, 4, 7, 10, 24, 30, 48, 72, 96, 144, and 168 hours were collected via jugular venipuncture. In addition, an oral administration of meloxicam at 1 mg/kg was evaluated as a control. Results presented in Fig 8 show that our method to assess meloxicam levels in bovine blood plasma was efficient. The control (oral) showed a pharmacokinetic behavior that is expected for meloxicam with the largest concentration in plasma after 24 hours (Fig 8B, orange line/Non-cumulative), showing that even at 160 hours there’s still meloxicam from that first dose [37]. Although microneedle patches reported a similar trend with the highest concentration around 24–48 hours) (Fig 8C and 8D, orange line/Non-cumulative), the amount released was minimal. In addition, the accumulative profile (blue line) (Fig 8B) for the oral administration presents a stationary release after 100 h, while the microneedle system (Figs 1D and 8C) still presents an increasing release after 100 h. These results validate the methodology here used to evaluate meloxicam levels in bovine blood plasma and motivate us to improve the patch to enhance the in-vivo transdermal drug release. Chitosan as natural biodegradable biopolymer has demonstrated have a degradation dependent on molecular weight, deacetylation degree, polydispersity, purity level, and moisture content [35]; therefore, these aspects of the chitosan may be considered. On the other hand, another crucial alternative may be focused on using hydrophilic polymers that help to solubilize the meloxicam. Hydrophilic polymers such as polyvinyl alcohol, polyvinylpyrrolidone, and sugars have been demonstrated to produce a fast capacity of dissolution [38]. Because meloxicam is a poorly water-soluble drug that only dissolves completely in a few solvents, such as dimethyl sulfoxide (DMSO) [21, 39], meloxicam needs to be in solubilized state [40].

## Conclusion

The results shown in this work may be viewed as the initial step in the fabrication of a biodegradable microneedle patch for livestock pain management with promising characteristics toward commercial manufacturing. A study of the rheological properties of the chitosan solution demonstrated that successful patches can be fabricated by reusing the solution for a maximum of 3 days, and deformed patches are obtained starting on the fourth day after solution preparation. Microscopy characterization revealed that microneedles with high concentration of the drug are uniformly organized on the patch surface and preserve their morphological properties after the sterilization process. FTIR spectra confirmed that microneedle patches with and without the drug preserve the chemical composition of chitosan polymer and meloxicam drug after the sterilization. Furthermore, full penetration of the microneedles into the skin can be obtained by applying approximately 3.2 N, and microneedles have the capability of returning to their original shape after compression testing. In-vitro drug release analysis reported that the microneedle patch provides a sustained release with approximately 33.02 ± 3.88% of the meloxicam released over 7 days. Additionally, the insertion study showed that microneedle patches were capable of dissolving in-vivo, exhibiting that the microneedle height decreases from 600 μm to less than 300 μm after penetrating the skin for 7 days. Finally, the area where the patch was inserted did not show any abnormal tissue, swelling or inflammation. Futures studies will be focused on studying the optimization of the transdermal drug capability of the microneedle patch. As chitosan is a natural biodegradable biopolymer which has demonstrated to have a degradation dependent on molecular weight, deacetylation degree, polydispersity, purity level, and moisture content [35]; these aspects may be considered to improve the in-vivo drug release capability of the patch. The results shown in this work may be viewed as the initial step in the fabrication of a biodegradable microneedle patch for livestock pain management with promising characteristics toward commercial manufacturing.

## Supporting information

S1 File A view of a complete assembly of patches and tapes to adherer microneedle patches to cow’s ear for the in-vivo studies. SEM image of a microneedle patch prepared with a 4-day old chitosan solution. SEM images of in-vivo degradation of different sections of the patch. Raw HPLC data for in-vitro drug release.
(DOCX)

S1 Graphical abstract (TIF)
