# Supplementary material for: Biodegradable microneedle patch for delivery of meloxicam for managing pain in cattle
Source: PLoS One. 2022 Aug 2;17(8):e0272169. doi: 10.1371/journal.pone.0272169 (PMC9345335; doi:10.1371/journal.pone.0272169)
Supplement: S1 File — SEM image of a microneedle patch prepared with a 4-day old chitosan solution. SEM images of in-vivo degradation of different sections of the patch. Raw HPLC data for in-vitro drug release. (DOCX) [file pone.0272169.s001.docx]

**Biodegradable microneedle patch for delivery of meloxicam for managing pain in cattle**

**David A. Castilla-Casadiego^a,b,1^, Katherine A. Miranda-Muñoz^c,1^, Jesse L. Roberts^a^, Anne D. Crowell^b^, David Gonzalez-Nino^d^, Dipankar Choudhury^e^, Frank O. Aparicio-Solis^a^, Shannon L. Servoss^a^, Adrianne M. Rosales^b^, Gary Prinz^d^, Min Zou^e^, Yuntao Zhang^f^, Johann F. Coetzee^f^,** **Lauren F. Greenlee^a^, Jeremy Powell^g^, and Jorge Almodovar^a*^**

*^a^Ralph E. Martin Department of Chemical Engineering, University of Arkansas, 3202 Bell Engineering Center, Fayetteville, AR 72701, USA*

*^b^Mcketta Department of Chemical Engineering, University of Texas at Austin, Austin, TX, 78712, USA*

*^c^Department of Biomedical Engineering, College of Engineering, University of Arkansas, Fayetteville, AR 72701, USA*

*^d^Department of Civil Engineering, University of Arkansas, 4190 Bell Engineering Center, Fayetteville, AR 72701, USA*

*^e^Department of Mechanical Engineering, University of Arkansas, 204 Mechanical Engineering Building, Fayetteville, AR 72701, USA*

*^f^Department of Anatomy and Physiology, College of Veterinary Medicine, Kansas State University, 228 Coles Hall, 1710 Denison Ave, Manhattan, KS 66506, USA*

*^g^Department of Animal Sciences, University of Arkansas, B110 Agriculture, Food and Life Sciences Building, Fayetteville, AR 72701, USA*

*^1^ D. A. C-C. and K. A. M-M. contributed equally*

**Corresponding author: Address* correspondence *to Jorge Almodovar, Ralph E. Martin Department of Chemical Engineering, University of Arkansas, 3202 Bell Engineering Center, Fayetteville, AR 72701, USA. Electronic mail:* [*jlalmodo@uark.edu*](mailto:jlalmodo@uark.edu) *Phone: +1 479-575-3924, Fax: +1 479-575-7926.*

**Supporting Information**

A view of a complete assembly of patches and tapes to adherer microneedle patches to cow’s ear for the in-vivo studies. SEM image of a microneedle patch prepared with a 4-day old chitosan solution. SEM images of in-vivo degradation of different sections of the patch. Raw HPLC data for in-vitro drug release.


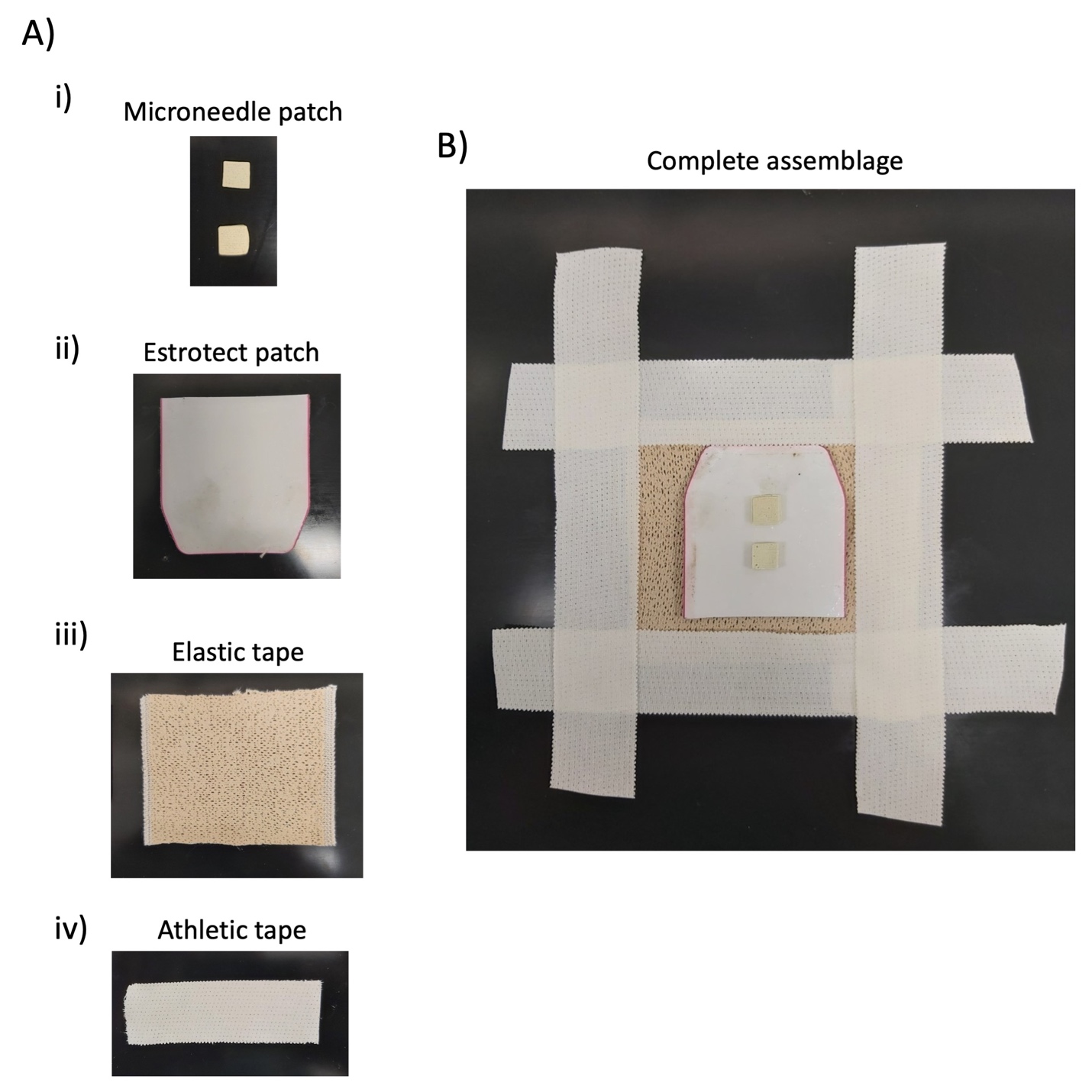


***Figure S1.*** *Patches and tapes. A) (i) Microneedle patches, (ii) estrotect patch, (iii) elastic tape, (iv) athletic tape, B) complete assemblage of patches and tapes.*

***
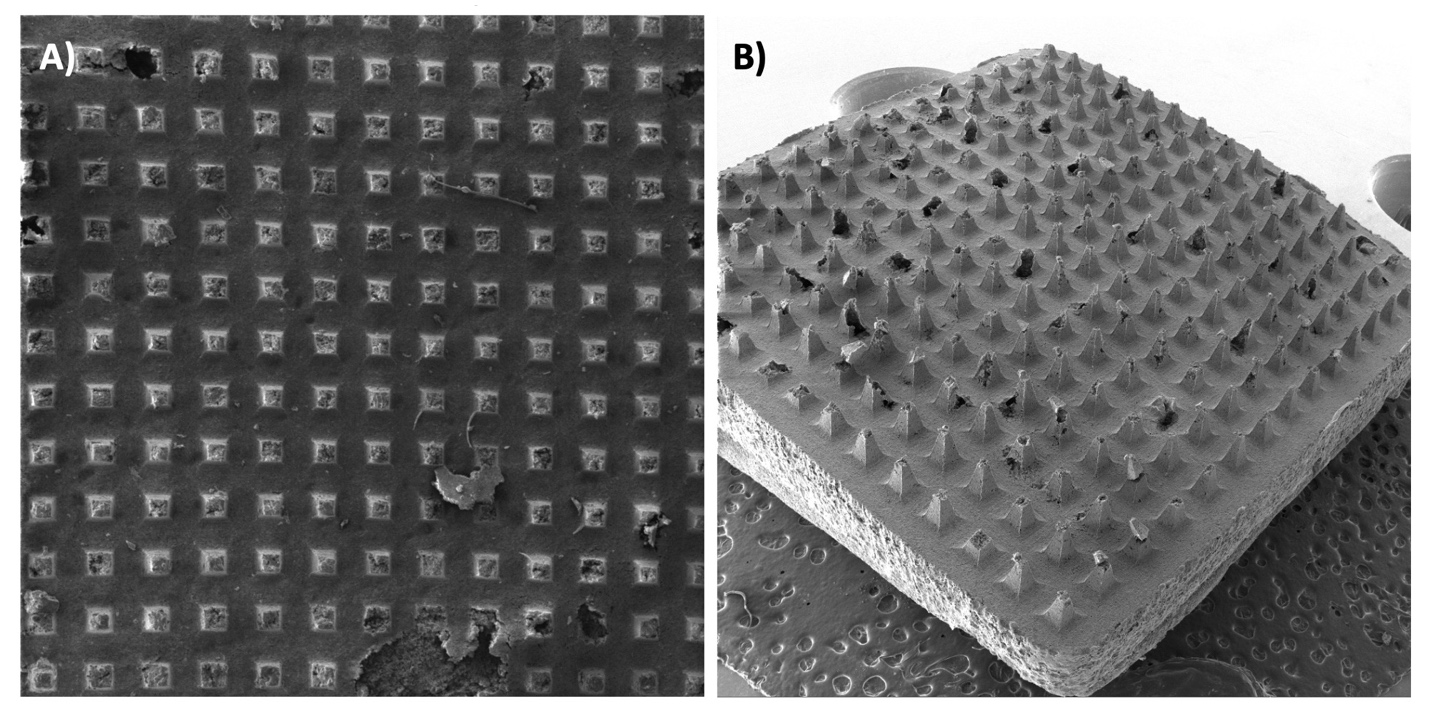
***

***Figure S2.*** *SEM image of a failure microneedle patches A) prepared with a 4-day old chitosan solution, B) Representative imagen of patches loaded with more than 125 mg of drug.*


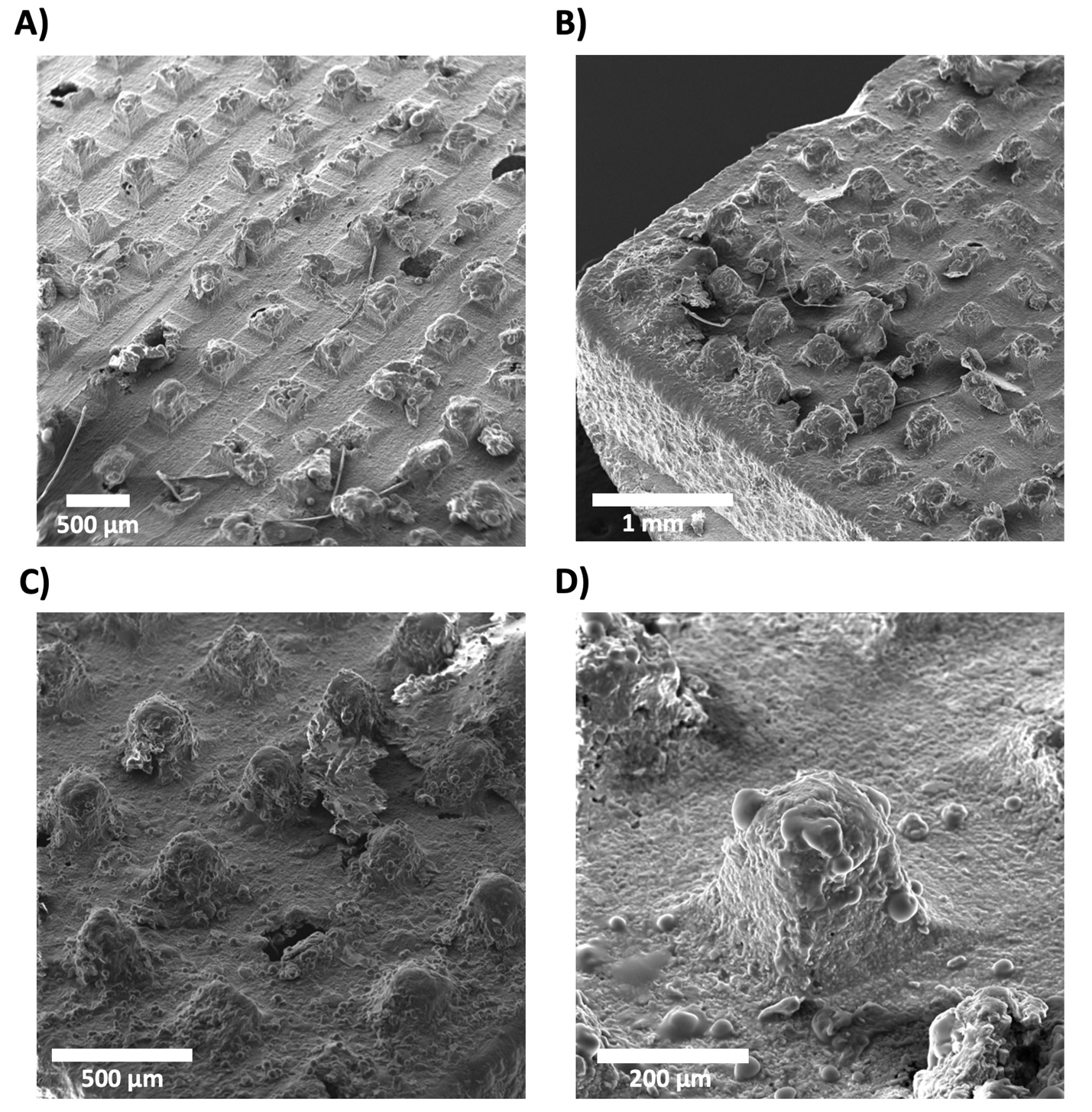


***Figure S3.*** *In-vivo degradation of different sections of the patch.*

***Table S1A.*** *In-vitro drug release raw data*

***Table S1B.*** *In-vitro drug release analysis*
