# Supplementary figures and images for: Biodegradable microneedle patch for delivery of meloxicam for managing pain in cattle
Source: PLoS One. 2022 Aug 2;17(8):e0272169. doi: 10.1371/journal.pone.0272169 (PMC9345335; doi:10.1371/journal.pone.0272169)

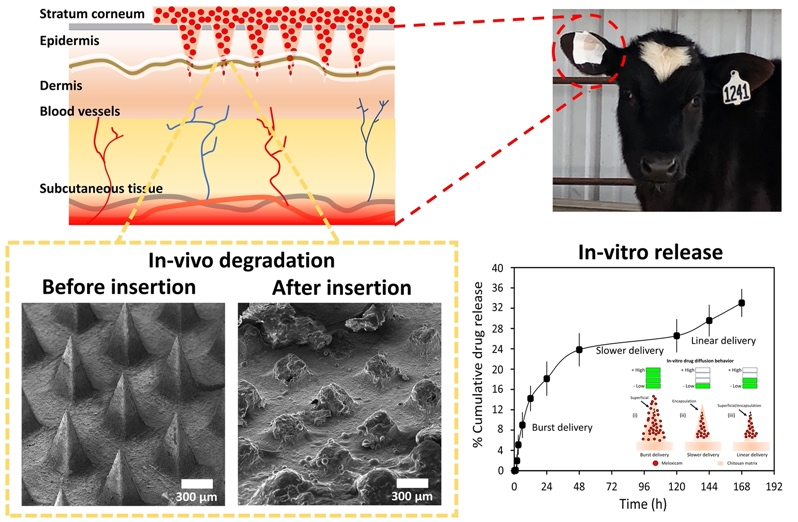

Supplement: S1 Graphical abstract — (TIF) [file pone.0272169.s002.tif]
